# Psychosocial support for survivors of violence against women: a qualitative study on provider and user perspectives in a Ugandan health facility

**Published:** 2026-01

**Authors:** Ruth Obaikol, Milton Mutto, Catherine Abbo, Michael Lowery Wilson

**Affiliations:** ^*a*^ University of Turku, Finland / Pincer Training and Research Institute, Uganda.; ^*b*^ Pincer Training and Research Institute, Uganda.; ^*c*^ Department of Psychiatry, School of Medicine, College of Health Sciences, Makerere University, Kampala, Uganda.; ^*d*^ Section for Oral Health, Heidelberg Institute of Global Health (HIGH), Heidelberg University, Im Neuenheimer Feld 130.3, 69120 Heidelberg, Germany.; ^*e*^ Injury Epidemiology and Prevention (IEP) Research Group, Turku Brain Injury Center, Turku University Hospital and University of Turku, Turku, Finland.

**Keywords:** Violence Against Women, Perceived Organization support, Primary Health Care, Health Care Worker

## Abstract

**Background:**

Violence Against Women (VAW) impacts 1 in 3 women worldwide, making it a significant public health problem. Most survivors will seek some form of care at healthcare facilities, often making hospitals a critical point of intervention. Psychosocial support plays a crucial role in the rehabilitation of survivors, helping them navigate the physical, emotional, and psychological consequences of violence. This study sought to assess the experiences of both Healthcare Workers (HCWs) and users of facility-based psychosocial care at a private, not-for-profit hospital in Uganda.

**Methods:**

A qualitative design using in-depth interviews was employed to explore experiences and perspectives of eight survivors and nine healthcare workers at a private not-for-profit hospital in Uganda in 2023.

**Results:**

The psychosocial services included screening, medical treatment, mental health support, referrals, and follow-up care. Key challenges identified were: limited Healthcare worker capacity to provide psychosocial care, inadequate infrastructure to provide safe spaces for care; high loss to follow up rate; and poorly formed networks with other service providers. While survivors appreciated care, findings emphasized the need for enhanced staff training, more tailored support for survivors and awareness creation for response services at the facilities.

**Conclusions:**

While survivors value psychosocial services, gaps remain in staff capacity, infrastructure, visibility, and follow-up. A client-centered approach that protects privacy, enhances training, and strengthens referral networks can make care more responsive, comprehensive, and sustainable for women affected by violence

## Introduction

**Global Burden**

Violence against women remains one of the most pervasive yet under-addressed public health problem, with far-reaching health and social consequences.^1^ It is defined as any act of violence that results in, or is likely to result in, physical, sexual or psychological harm or suffering to women, including threats of such acts, coercion or arbitrary deprivation of liberty, whether occurring in public or private life.^2^ Globally, the lifetime prevalence of physical and/or sexual VAW is estimated to be 1 in 3.^3,4^ In sub-Saharan Africa, a systematic review from 2008 to 2019 revealed majority of women suffered emotional violence (29.40%), followed by physical violence (25.87%) and sexual violence (18.75%) ; East Africa was particularly highly burdened with reported emotional Violence ( 25%).^5^

As a high-income country, the United States of America (USA) is severely dealing with this challenge, so in 2011, more than 32,000 deaths were recorded as being caused by PVF.

**Health consequences**

Violence against women is associated with a wide range of negative effects. These include physical effects such as acute injuries and chronic pain ; sexual and reproductive health issues including abortions, chronic pelvic pain, and sexually transmitted infections; and mental health issues like post-traumatic stress disorder (PTSD), anxiety disorders, substance abuse, and suicidal behavior.^6^ As a result of its impact on women’s health, VAW accounts for a significant number of health facility visits.^7,8^ Unless these consequences are addressed adequately, survivors may survive with sub optimal quality of life.^9^

**Role of Health systems**

Addressing VAW in health care system requires multidisciplinary and comprehensive approaches.^10,11^ The primary goal of these services is to ensure holistic care, addressing physical, mental and social well-being of patients. Current healthcare designs for psychosocial care ensure an integrated psychosocial package alongside medico-legal services for survivors. These services should ideally include; medical treatment of VAW related physical trauma to protects them from further injury; HIV counselling and testing, post-exposure prophylaxis(PEP) for HIV, and contraception for survivors of sexual assault; counselling to address immediate mental health issues; medicolegal services and community referrals for appropriate reintegration into society.^12,13^ Despite the clearly known and documented roles of HCWs towards addressing VAW in health systems, there is still limited published information regarding general progress in adopting standards and practices, and their effectiveness in facilities in sub-Saharan Africa. 

**Challenges in sub-Saharan Africa and Uganda**

The limited available evidence indicates that significant challenges persist, particularly in Africa. For example, in Kenya, patient engagement remains generally low, with fewer than 20% of clients returning for a second counselling session. Studies also reveal that healthcare workers often lack the capacity and time to address patients’ psychological and emotional needs.^14^ Similarly, qualitative research in Blantyre, Malawi, highlighted that providers felt inadequately prepared to handle the psychosocial and mental health consequences of women who were survivors of violence compounded by staff shortages and a lack of clarity regarding the health sector's mandate and the definition and need for ‘counselling’.^15^ Further still, a 2020 scoping review of 54 studies from East Africa revealed that the current models of psychosocial service provision seem to be inadequate to meet the medical and psychosocial needs of survivors, defining the need to explore policy implementation.^16^

**Study rationale and objectives**

In the Ugandan context, many studies primarily address the burden of VAW in emergency situations, despite a high prevalence of VAW in the general population of 56% married women aged 15-49 years.^17^ Moreover, the provision of routine mental health care within public health systems is hindered by substantial challenges, including inadequate staffing, limited training capacity, and persistent barriers to service access.^18^ Consequently, providing psychosocial care within the health care framework could be even more challenging given the context and the constructs surrounding VAW. Despite these issues, there is a limited information regarding provider and user experiences; and available services within the Ugandan context. A qualitative survey in 2018 explored barriers and enhancers of implementation of international guidelines in Uganda however, it did not provide in-depth exploration of HCWs and user perspective nor document actual services that were provided in the health facilities.^19^

This study aimed to explore psychosocial services available for VAW survivors at an urban hospital in Kampala-Uganda, and assess the user and provider experiences to identify service strengths, gaps, and recommendations.

## Methods 

**Participants**

The research participants included eight survivors of violence, aged between 18 and 64 years, who had received services between February and March 2023. Participants were selected using purposive sampling. The majority (7/8) had survived sexual violence, while one had experienced physical violence. The study also included ten healthcare workers (HCWs) who provided care at stations managing survivors of gender-based violence (GBV) during the study period. These included counsellors at the HIV/AIDS clinic, a psychiatric nurse, a paramedical worker, a psychiatrist, medical officers (doctors), and general nurses (see Table 1). 

**Table 1:** showing characteristics of Health Care worker participants

HCW type	No	Gender
Psychiatric Nurse	1	F
Psychiatric clinical officer	1	M
Counsellors	3	F
GBV focal person	1	F
Medical Officer	1	M
Psychiatrist	1	F
Manager General Out Patients	1	F

Each interview lasted approximately 45minutes to 1 hour. Data saturation was achieved by the eighth survivor participant, at which point no new information regarding the facility’s services emerged. All HCWs directly involved in the provision of psychosocial care to survivors were included in the interviews.

**Study Setting**

The study was conducted in a non-profit hospital located in Kampala, Uganda’s capital city. The hospital is a key provider of psychosocial services for GBV, offering a comprehensive range of healthcare services, including mental health care. Its mental health services were administered by a psychiatric nurse and a clinical officer, with the support of a visiting psychiatrist available twice a week. Both inpatient and outpatient mental health services were offered, with complex psychiatric cases referred to national referral hospitals within a 10 km radius.

**Data Collection and Analysis**

Interview data were captured using audio recorders and transcribed verbatim. The transcripts were anonymized. Data analysis was conducted using "NVivo 14 Lumivero." Codes were generated through both deductive and inductive approaches. The deductive approach involved analyzing data based on literature reviews, identifying trends in practices, and considering the views of VAW survivors and HCWs. Additionally, the inductive approach was applied by analyzing the data broadly based on the respondents' submissions. Themes were generated using content analysis to document the services provided, challenges faced, perceptions, and recommendations from HCWs and VAW survivors.

## Results

We carried out a total of 17 in-depth interviews, of which 8 were with female survivors of violence against women (VAW) who had accessed services in the hospital between February 2023 and March 2023, and 9 HCWs. The HCWs who typically interacted with VAW survivors at the out patients were included both men (2/9) and women (7/9). Participants were between the ages of 22 and 45 years. The HCWs included; General Out Patient Department (GOPD) staff, psychiatric clinic staff and the HIV/AIDs clinic Staff.

**Services**

The findings revealed that the hospital received patients from a broad geographical area beyond its immediate catchment. The majority of cases were referred by friends or community members familiar with the hospital's services. However, none of the participants were aware that the hospital provided psychosocial services for clients exposed to VAW before seeking care.

Participants described a wide range of services available at the hospital (see Figure 1). Survivors seemed to have gained confidence in the facility throughout their treatment process. Most survivors, being survivors of sexual violence, were primarily concerned about acquiring HIV. Consequently, their main motivation for seeking health services was to access prevention services.

**Figure 1:**
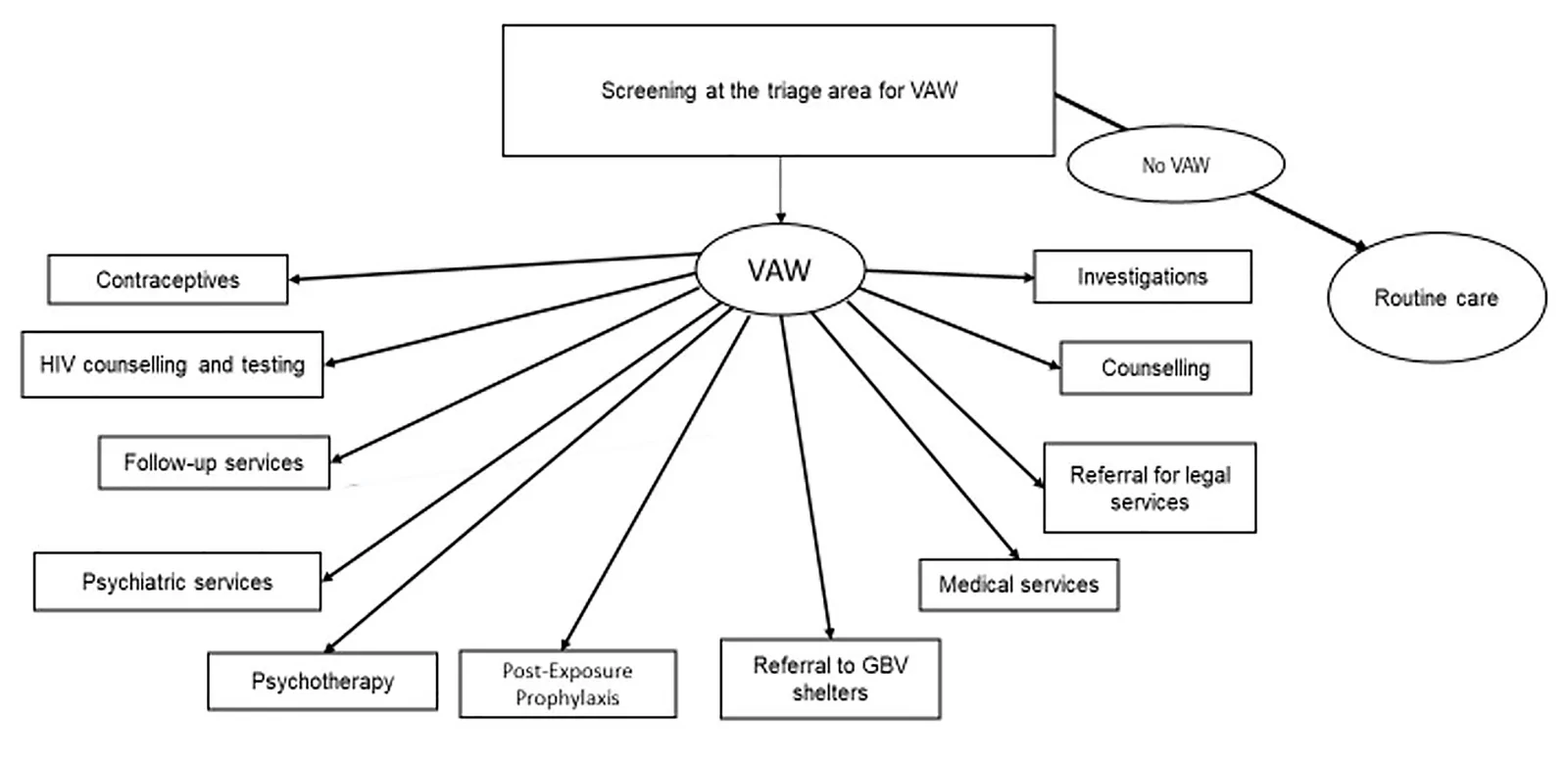
Hospital Services for VAW Survivors The figure illustrates a comprehensive set of services offered to survivors of VAW at the hospital. Upon screening at the triage area, patients were assessed for VAW, mental health effects, and other health concerns. If WAW was identified, the patient is provided with a range of services, including medical treatment, psychological counselling, contraceptives, HIV counselling, post-exposure prophylaxis, and psychiatric care. Additional services include referrals, for legal, other GBV services and follow-up care.

One of the participants for example mentioned having been referred by a workmate to the hospital having suffered sexual violence; “So, Monday morning I shared my story with that guy my colleague and he is the one that told me about this Hospital. He told me that he had a sister working in this hospital that could help me. So, he called his sister, I went to This Hospital and I was able to receive the care I needed’’ KII VAW survivor

Another client who had suffered physical violence was unaware of the services offered and came to the hospital solely due to her physical injuries. She did not know that she could also receive psychosocial care following a VAW event. ‘’We also just found these services here. Hadn’t we come, we would never have known’’ KII VAW survivor

**Screening**

Although none of the VAW survivors described screening as their entry point to care, HCWs reported that screening for GBV was the first stage in providing psychosocial support to VAW survivors. The hospital had a triage area dedicated to these services, equipped with a screening tool and staffed by a designated psychiatric nurse. The nurses, trained and experienced in mental health and GBV, were responsible for screening all individuals attending care at the hospital to identify survivors. Screening a client at the triage seemed to provide an opportunity for them to access different services at the facility depending on their presentation “I majorly do assessments of patients at the triage, at the screening point and refer them to the metal health department and the different sectors within the hospital”. KII – HCW 

According to the HCWs, the screening process for VAW and its outcomes seemed to be very uncomfortable for the survivors. Most survivors appeared reluctant to volunteer information about the violence they had experienced. As a result, HCWs often had to probe further to obtain information from clients who exhibited signs of potential abuse;

 “At the triage, we have a psychiatric nurse who sits there and she is always able to probe and see that however much I’m dealing with this person, he or she is going through a lot. That’s how we have been able to capture the rape cases and those that have been abused by their husbands. But it’s very important to note that it’s very difficult to identify them because they hide. They do not come out due to fear of stigma. We normally get them when they present with extreme conditions like for example yesterday, we got two cases that had taken poison. This was a husband that was brought in by the wife. And they had taken poison. The wife was the one now telling us what had happened. But from the story you could see that there was spark” KII – HCW

It appeared that having a psychiatric nurse at the screening area improved the screening for mental health illnesses. Findings reveal that screening included identification of the causes of violence to institute appropriate treatment. Additionally, the nurse was able to link the clients to appropriate care;

 ‘’…but you are also identifying the cause of the violence. If its alcohol and drug abuse, you are going to deal with that. If its psychotic you put them on either treatment or they are referred’’. KII – HCW 

Sometimes cases of VAW would bypass the initial triage process and be identified by medical personnel later on. One respondent reported instances where VAW cases requiring psychosocial support would be identified during clinical examinations or through internal referrals. Additionally, health education played a significant role in the hospital, as the respondent noted that medical talks created awareness and increased demand for services;

 “Usually at the triage area, the psychiatric nurse sits there. So, I think they ask a few questions, like what has brought you, so they can direct and push you to the right department. Number two, they can be identified through the screening process once they have gone to see a doctor. The doctor will identify and say this is not medical but rather psychiatry. But there are those who will come specifically knowing what they want. And then there are those who are referred based on the medical talks or briefs that are held here every morning.” KII - HCW

Although the hospital had checklists for screening for GBV, interviews revealed that these tools were not widely adopted by the team because they did not know that the tool existed. Some healthcare service providers used the patient history to guide the assessment of the identified survivors. They also used standard psychiatric tools like DSM5 to facilitate the diagnosis process for any mental health outcomes;

 “Personally, what I know is we don’t have an assessment tool to do that. It’s basically the patient history that is going to be used to determine that maybe someone was assaulted at home the last time and so you probe further. Otherwise, the practice of using a check list, we don’t.” KII HCW

**Medical Interventions**

Management of VAW by the HCWs generally involved a full clinical examination, investigations and appropriate interventions; Fore-example if a survivor was physically hurt, the physical trauma was managed, if they were sexually assaulted they were investigated for HIV. Treatment was instituted based on findings. Post-exposure Prophylaxis and Contraception was always provided for those who had experienced sexual assault. 

 ‘’So, no one really helped me apart from the fact that I came here to this hospital and sought treatment. I was able to prevent myself from getting pregnant and contracting HIV”. KII VAW survivor

 ‘’I felt very bad of course and I was worried but I had to share what had happened to me with one of my friends. So, they are the ones that told me about that medication called PEP and where I could get it. So, I had to rush to the hospital the following day very early I the morning. So actually, what happened is I went to the hospital that evening they gave me one PEP tablet and told me to come back the next morning’’. KII VAW survivor 

 ‘’It’s not much because majority of them will show up if they have been physically hurt to receive care. So, it basically treating the wounds and bruises caused by the violence and also, we try to ensure that the internal organs were not affected. Then we also provide pain killers mainly.’’ KII HCW

 ‘When they come, they go through our triage area. At that triage area, we have a psychiatric nurse who is able to accurately identify these individuals. She first ensures they receive general medical care, especially for any injuries, which are treated before they are referred to the mental health clinic here”. KII HCW

**Mental Health Services**

The hospital appeared to offer both basic and specialized mental health services. These included counselling for survivors’ mental health support, counselling for HIV testing, and psychiatric services for survivors. The majority of survivors reported receiving adequate counselling. However, some indicated that they received only "some sort of advice," suggesting that they expected more comprehensive support than they received;

 ‘’When I got to the reception at the counselling department, the receptionist asked me whom they were going to test and I told her it’s my children. So, they sent me to a room where I would meet counsellor number four. I went there with a lot of fear in me wondering why I was being sent to that specific room yet they haven’t even tested my children. When I got there, I was warmly welcomed and counselled very well’’. KII VAW survivor 

 ‘’So, I was registered, they first took a blood sample from me for testing. This came out negative and I was ok. So, they also gave me oba (maybe) I can call it advice?’’ KII VAW survivor 

Findings further revealed that despite the hospital's good reputation in handling mental health-related cases, gaps in mental healthcare service delivery existed. Healthcare worker respondents noted a lack of a psychologist and a dedicated counsellor specifically for mental health. Present counsellors in the hospital primarily focused on survivors of sexual assault and HIV, and none were trained to provide professional counselling services. Counselling was primarily handled by the psychiatrist, clinical psychiatric officer, or psychiatric nurse, while severe cases were referred out. This limited the counselling services provided by the hospital to what could be managed by the available staff. Additionally, it was noted that specialised mental health services were available on a part-time basis, leading to service gaps on certain days;

“We do provide both in-patient and outpatient services but mainly outpatient services. It’s a department with three people, me as the psychiatrist, PCO and then we have a psychiatric nurse. Personally, I come twice a week and the PCO comes the rest of the days except on weekends unless if there is an emergency or a scheduled appointment. For the outpatient we receive and treat anyone on an out-patient basis”. KII HCW

 ‘When you look at mental healthcare, we have the treatment and the therapy. This is because we have the chemotherapy which is the medication and the psych therapy which involves seeing the psychologist for counselling. There are these other modalities. They are so many but these are the two commonly offered here at this hospital. Now in regards to the psych therapy, the psychologist we refer to goes as far as meeting the relatives’’. KII - HCW

 ‘’You see to be very fair and I don’t want to undermine any one, I think they are mainly trained in HIV counselling and it has nothing to do with this mental health care department. They are not qualified to do psychiatric counselling’’. KII HCW

**Referral Services**

Health care workers reported referral of clients for legal services, to GBV shelters and for further mental health care services. Some survivors however indicated no confidence in legal services and were not interested in pursuing them further;

‘’Just like any other hospital that you can run to, you can find help and a dedicated person. I didn’t mind about going to police because it wasn’t going to help in any way. They would still have to refer me to a hospital to get the PEP. I even remember when I came here a lady asked me whether I wanted to take the matter to the police and I said no. This is because it’s me that accepted to go and sleep at his place. Had I not accepted to go with him, none of that would have happened’’ KII HCW 

**Follow-up Services**

Healthcare workers acknowledged the need for follow-up services, which typically involve scheduling appointments for clients. However, it was reported that clients rarely returned for these follow-ups. To address this gap, some healthcare workers provided follow-up services by phone. Others seemed to be constrained by time and resources. Some healthcare workers indicated that follow-up services need to be client-centered. One respondent noted that follow-up could only be successful if it aligned with the survivor’s interests. Survivors of sexual violence for example appeared primarily interested in follow-up for HIV test results. One survivor indicated that, since the perpetrator tested negative and had apologized, she felt relieved and forgave him, seeing no further need for follow-up care.

‘’ I will be honest with you; I don’t think its possible basing on the work that we do. We have a lot of clients to attend to and we are not facilitated to be able to follow up’’. KII – HCW

 ‘’…. I’m supposed to go back on the 28th march 2023. So, I haven’t yet gone back. I wanted to go back and tell them that I stopped the medication. It was making me feel not so good. The fact that I went and tested with the perpetrator and he turned out negative, he apologized. You know we as women have soft hearts so I forgave him. But all in all, I was happy that he was negative’’. KII VAW survivor 

‘’The follow ups are positive if the patients are ready to take on the support. But you normally find some people that aren’t ready. For example, in case of a rape case or something, some other people might not want to go that far. Some prefer separating, doing other things. But in most cases, we want the idea of this sort of support to come from them. This is because if you imposed an idea on someone, they are likely to reject it. That’s what I’m thinking. But if you allow them to come up with their own ideas on how they can improve their situation, then it will work in most cases’’. KII HCW

**Nature and Quality of Services**

Majority of the participants appreciated the services they had received at the health facility. They reported good, professional, quick and compassionately delivered services. Some reported lack of privacy. The cost of services was said to be free or affordable. Survivors recommended more awareness of the services in the community, better counselling services, couple counselling and separation of VAW services from HIV care. 

 ‘’The benefits of seeking care here are that they work on patients very fast. They will welcome you and make you feel at home. But what they should improve is that they should have in place; a private room where each and every one of us first go into that room and receive counselling. Not mixing VAW survivors with HIV and other mentally ill patients. But they generally have no problem’’ KII VAW survivor

‘’They handled me so well and they were professional’’ VAW survivor 

‘’They should organize awareness campaign of VAW and mental health generally. This information should be out there because there are a lot of people suffering in silence and don’t know where to go. They should also equip us with vocational skills so that we aren’t dormant.’’ KII VAW survivor 

‘’…. I think the health workers at least should be a little more caring because people in my state are already angry and have lost hope. So, in order to bring back that hope in them I request that they should also improve on the counselling because other patients are still coming. People are still being abused out there and there are those that can’t afford to come to this place. Some don’t have transport; some cannot manage the money for the scan. Some might not be in position to see a doctor. Yet they are being abused and would like access to your services. So, we request that people are helped in that regard’’. KII VAW survivor

‘’My suggestion is that in case a case of abuse is brought and its between partners, both people should be invited for the counselling sessions. This is because when both sides are heard, it is easier to solve the problem. This is because normally when you listen to one side of the story, you may not be able to hear from the other side. This might complicate the healing process’’ KII VAW survivor.

## Discussion

The study revealed that the hospital delivered a variety of psychosocial care services within it’s expected scope as outlined by the national guidelines for psychosocial care. Clients seemed to be satisfied with the care provided by the hospital and found it to be professional and empathetic. Services included medical treatment and prevention of HIV and unwanted pregnancy, mental health care, and referrals for other services such as legal aid and specialized mental health care. Although psychosocial services were partially integrated into mainstream hospital functions, they were primarily provided through the HIV clinic. Being largely donor-dependent and focused on sexual violence left healthcare workers without adequate skills to address broader psychosocial needs. Consequently, survivors of physical abuse who do not access services through the HIV clinic do not seem to appreciate the psychosocial support provided. This finding aligns with numerous studies showing that HCWs without a mental health background often feel incompetent to provide psychosocial care. For example in Jamaica while midwives were aware of VAW, they reported an inability to respond effectively due to a lack of proper training.^20^ Furthermore, although Kanagasabai et al. describe an increase in GBV facilities in Sub-Saharan Africa through the President’s Emergency Plan for AIDS Relief(PEPFAR) project; outside of well-funded HIV services, mainstream GBV services may still face significant challenges.^21^

Screening for VAW and mental health issues posed challenges for HCWs and survivors due to inadequate levels of privacy in the traditional triage areas. The sensitive nature of the screening questions made HCWs uncomfortable, and they recommended the need for more privacy. This could further be worsened by the fact that VAW is often compounded by stigma. The fear of judgment, social exclusion, or blame may limit how they interact with service providers, which can prevent survivors from seeking care or disclosing their experiences. This aligns with findings in Tanzania and South Africa were screening services necessitated confidentiality, staff empathy, and the absence of judgment.^22^ Medical treatment seemed to serve as an entry point into psychosocial services for some survivors. This aligns with findings indicating the significant contribution of VAW to outpatient visits and the necessity of offering screening services at health facilities in Asia and Europe.^23^ Although comprehensive care is recommended for survivors, in some cases, basic medical care appeared to be sufficient for survivors. For instance, clients who had experienced sexual assault seemed to be satisfied with HIV counselling, testing, and pre-exposure prophylaxis (PREP) services. Receiving a negative HIV test result provided a sense of relief, reducing their need for further follow-up services. However, some survivors still wanted linkage to legal services, highlighting the importance of providing a comprehensive range of support services. This underscores the need to offer integrated and accessible services to meet the diverse needs of VAW survivors. 

Healthcare Workers seemed to be constrained in providing care due to inadequate staffing, insufficient infrastructure, and lack of capacity. This aligns with findings from the qualitative National Survey on Violence in Uganda conducted in 2020. The study by the Uganda Bureau of Statistics revealed that, despite the critical ramifications of VAW, the issue was undermined by a lack of accessible and integrated services, poor reporting mechanisms, and weak institutional capacity across sectors.^19,24^

Follow-up services seemed to be underutilized. Many clients, after receiving initial medical treatment, perceived no further need for continued care. For others requiring additional counselling sessions, the financial burden deterred them from seeking follow-up services. Although some providers went beyond routine practice by initiating follow-up calls, HCW reported that follow-up efforts were largely contingent upon the clients' willingness to engage. Overall, the follow-up process appeared to be client-driven. In contrast, evidence from some studies suggests that group therapy can improve follow-up adherence. For instance, a pilot study on group compassion-based therapy among Colombian female survivors demonstrated a retention rate of up to 89% after three months.^25^ This may imply that survivor-clients receiving care at the hospital may not be utilizing follow-up services because these services are not designed to meet their needs; yet follow-up is critical because it ensures continuity of care, addresses ongoing psychosocial needs, and helps prevent further harm or complications.^26^

**Policy Implications: **Based on insights from this study, several considerations emerge for policy and practice:

• The Ministry of Health in Uganda could explore the mainstreaming of GBV services across different departments and consider building the capacity of healthcare workers to respond effectively, as GBV cases may present in diverse clinical settings.

• Health facilities might find it useful to adapt national guidelines into context-specific protocols that align with their workflow and facility design, given the challenges observed in translating standard guidelines into practice.

• Donor agencies could consider supporting integrated service approaches in resource-limited settings, as participants suggested that such strategies may improve sustainability and address multiple health needs simultaneously

## Conclusion

Survivors of violence against women (VAW) appeared to be confident in facility-based psychosocial care. However, the delivery framework must account for the specific needs of survivors, the capacity-building requirements of HCWs, the design of supportive infrastructure, mechanisms for integration with other psychosocial service providers, and the visibility of these services within the community. Despite being situated in an urban environment with access to formal mental health care services, the hospital under study continues to face significant challenges in delivering effective psychosocial support. These findings suggest that the challenges may be even more pronounced in less advantaged or resource-limited settings. Further research is necessary to identify strategies for optimizing the delivery of psychosocial services in health facility contexts to address critical gaps, such as the lack of client-sensitive standardized screening tools to identify survivors’ needs, insufficient numbers of trained and skilled professionals to provide timely and appropriate psychosocial support, limited awareness among survivors of available facility-based services, and inadequate integration of psychosocial care into routine clinical workflows.

**Acknowledgment**

The authors express their sincere appreciation to the staff of the participating health facility for their invaluable support, and to the research assistants, Henry Bazira and Rhoda Topacho, for their dedication and commitment to the study. We are especially grateful to the study participants for generously sharing their time and experiences, without whom this research would not have been possible. 

**Authors’ Contribution:**

RO conceived and led the study, served as the corresponding author, and took primary responsibility for drafting and revising the manuscript.

MM critically reviewed the manuscript and provided technical guidance on violence and injury-related content.

CA contributed to the contextualization of the paper and provided expert input on psychosocial and cultural dimensions.

MLW contributed to the conceptual framing, overall supervision, and final review of the manuscript as the senior (last) author.

All authors read and approved the final version of the manuscript.
